# Habitat disturbance and the organization of bacterial communities in Neotropical hematophagous arthropods

**DOI:** 10.1371/journal.pone.0222145

**Published:** 2019-09-06

**Authors:** Kelly L. Bennett, Alejandro Almanza, W. Owen McMillan, Kristin Saltonstall, Evangelina López Vdovenko, Jorge S. Vinda, Luis Mejia, Kaitlin Driesse, Luis F. De León, Jose R. Loaiza

**Affiliations:** 1 Smithsonian Tropical Research Institute, Balboa Ancon, República de Panamá; 2 Instituto de Investigaciones Científicas y Servicios de Alta Tecnología, Panamá, República de Panamá; 3 University at Albany, State University of New York, NY, United States of America; 4 Department of Biology, University of Massachusetts Boston, Boston, MA, United States of America; 5 Programa Centroamericano de Maestría en Entomología, Universidad de Panamá, Panamá, República de Panamá; University of California San Diego, UNITED STATES

## Abstract

The microbiome plays a key role in the biology, ecology and evolution of arthropod vectors of human pathogens. Vector-bacterial interactions could alter disease transmission dynamics through modulating pathogen replication and/or vector fitness. Nonetheless, our understanding of the factors shaping the bacterial community in arthropod vectors is incomplete. Using large-scale 16S amplicon sequencing, we examine how habitat disturbance structures the bacterial assemblages of field-collected whole-body hematophagous arthropods that vector human pathogens including mosquitoes (Culicidae), sand flies (Psychodidae), biting midges (Ceratopogonidae) and hard ticks (Ixodidae). We found that all comparisons of the bacterial community among species yielded statistically significant differences, but a difference was not observed between adults and nymphs of the hard tick, *Haemaphysalis juxtakochi*. While *Culicoides* species had the most distinct bacterial community among dipterans, tick species were composed of entirely different bacterial OTU’s. We observed differences in the proportions of some bacterial types between pristine and disturbed habitats for *Coquillettidia* mosquitoes, *Culex* mosquitoes, and *Lutzomyia* sand flies, but their associations differed within and among arthropod assemblages. In contrast, habitat quality was a poor predictor of differences in bacterial classes for *Culicoides* biting midges and hard tick species. In general, similarities in the bacterial communities among hematophagous arthropods could be explained by their phylogenetic relatedness, although intraspecific variation seems influenced by habitat disturbance.

## Introduction

Bacterial communities are important components of hematophagous arthropods (e.g., blood feeders) vectoring disease-causing pathogens to humans and wildlife, and they are likely to play a key role in vector ecology, evolution and transmission capacity [1–4]. Several important human and animal diseases result from bacterial infection transmitted through the bite of arthropod vectors [5,6]. Bacteria also interact with the arthropod host to reduce or increase the transmission of pathogens or indirectly alter disease dynamics through the modification of nutrition [7], development, reproduction or the immune response of arthropod vectors [8,9]. Our understanding of the factors shaping the organization of bacterial communities in hematophagous arthropods vectoring human diseases is still limited. Studies regarding the microbiome of disease vectors have attempted to describe the structure and bacterial composition of specific taxonomic groups of arthropods, and to understand how it varies according to particular ecological or physiological factors, with the most comprehensive studies focused on mosquitoes [10] and ticks [2]. Although some studies have considered the impact of habitat or environment type on arthropod microbiota in mosquitoes [11–16], ticks [17–20] and biting midges [21], none to date have investigated the role of habitat disturbance in shaping bacterial assemblages among co-distributed hematophagous arthropods.

Mosquitoes (Diptera: Culicidae), sand flies (Diptera: Psychodidae), biting midges (Diptera: Ceratopogonidae) and hard ticks (Acari: Ixodidae) are collectively responsible for numerous medically important diseases worldwide, including arthropod-borne viruses (e.g., arboviruses) transmitted to humans (Dengue–DENV, chikungunya—CHIKV, Zika—ZIKV, Yellow Fever–YFV, West Nile–WNV, Mayaro and Oropuche) and to agriculturally important livestock (Vesicular Stomatitis–VSV, Blue-Tongue–BTV, Epizootic Hemorrhagic Disease–EHDV and African Horse Sickness–AHSV) or to both (Venezuelan Equine Encephalitis–VEEV, Eastern Equine Encephalitis–EEEV and Rift Valley Fever) [22,23]. In addition, some species in these arthropod assemblages are involved in the transmission of parasites such as filarial nematodes (*Mansonella—*filariasis) [24], protozoan (*Leishmania—*Leishmaniasis) [25] and bacteria (*Rickettsia*—Lyme disease and babesiosis) [26].

The ability of hematophagous arthropods to carry and transmit pathogens biologically is given by their population dynamics and feeding behaviour in relation to that of their vertebrate host, plus their immune responses to infection [9,27]. Some bacterial commensals impact the capacity of arthropods as biological vectors, through diminishing pathogen replication and dissemination in the host tissues or by reducing vector fitness and lifespan [4,9,28,29]. Studies from members of the Culicidae demonstrate the importance of the microbiome in modulating disease transmission. For example, *Chromobacterium*, *Proteus* and *Paenibacillus* bacteria can inhibit DENV replication in mosquitoes while the resident bacteria are required for its establishment [28]. Furthermore, the intracellular bacterium *Wolbachia* is known to adversely influence the transmission of DENV, CHIKV, ZIKV, YFV and WNV [30–35]. Alternatively, some bacteria are associated with an increase in disease transmission by their arthropod vectors. For example, members of Enterobacteriae are correlated with higher *Plasmodium* infection rates in *Anopheles* mosquitoes, while *Serratia odorifera* can increase the replication of both DENV and CHIKV in the midgut of *Aedes aegypti* [28,36]. Although, studies have endeavored to characterize the core microbiome of members of Psychodidae sand flies, Ceratopogonidae biting midges and Ixodidae hard ticks, it is still generally unknown how similar or different their microbiomes are, and whether some bacteria may influence disease transmission dynamics in these arthropod assemblages [18,19,21,37–42]. Nonetheless, some studies have revealed that resident bacteria are essential for the development of *Leishmania* parasite in Psychodidae through antibiotic treatment [37,43].

Metagenomic studies of disease vectors in the Order Diptera have revealed that different genera including those with a distinct ecology generally share a core microbiome, but often exhibit differences in bacterial composition and structure that distinguish a species [1,13,38,44–46]. Conversely, tick species may exhibit a distinct taxonomic structure in their microbiome, because they are associated with specific vertebrate hosts throughout their entire lifetime, including during the immature stages [47]. Core microbiota of Diptera are largely acquired from the environment during the immature stages, many of which persist until the adult stage [13,48–50]. Bacteria are also acquired during adult blood feeding, therefore the microbiome of arthropod vectors is likely impacted by both developmental stage and gender [1,19,49,51]. The core microbiota of ticks is either maternally-inherited, acquired from blood feeding on hosts or through the colonization of environmental microorganisms from vertebrate skin or the soil on physical contact [2,52].

Hematophagous arthropods can exhibit intra-specific variation in their bacterial associates between geographic locations, explained by differences in the quality of larval habitats or host preferences at sampling sites for both Diptera [11,37,46,53] and ticks [17,18]. Hence, it has been proposed that larval habitat conditions and geographic location are important factors shaping the bacterial community of some adult hematophagous arthropods. Conversely, some mosquitoes [44] and ticks [54] do not exhibit intra-specific variation in the bacterial community across geographical locations or habitats. This finding supports a more specific and long term association between some blood-feeding arthropods and their bacterial associates, which is likely mediated by the immune system of the host, rather than by their external environment [13,54].

Our goal herein is to test for variation in the diversity of bacteria among four epidemiologically discrete groups of hematophagous arthropods, and to identify the factors shaping this variation. Specifically, we address the following questions: (1) *How do patterns of bacterial diversity and composition differ among the microbiomes of mosquitoes*, *biting midges*, *sand flies and hard ticks*?, and (2) *Does habitat disturbance influence the organization of bacterial communities within these arthropod assemblages*? We posit that blood-feeding arthropod species in the Order Diptera will harbor comparable bacterial organizations, since they are more closely phylogenetically related, while hard ticks within the Order Ixodida are considered as an outgroup. We also postulate that intra-specific bacterial diversity and taxa composition will change owing to variation in habitat quality, but changes are only expected within Culicidae mosquitoes, Psychodidae sand flies and Ceratopogonidae biting midges. This is anticipated because host–tick interactions in obligated ectoparasites such as hard ticks are more likely to shape their microbiome than habitat disturbance. Although hard ticks can acquire surface microbiota from their environment, our study largely targets intracellular and gut bacteria colonized through vertical transmission or ingestation. We use a metabarcoding approach to compare inter-and intra- group bacterial communities among these arthropod assemblages, and also in relation to changes in habitat quality. If habitat disturbance is a significant predictor of bacterial assemblages, this could have ramifications for disease transmission through variation of the vector microbiome and correlated vectorial capacity.

## Materials and methods

### Arthropod collection and sample preparation

Permission was obtained from MiAmbiente under permit identification ID 8-447-900-PAN. The study was conducted in the lowland tropical rainforest ecosystem of central Panama, a region formerly known as the Panama Canal Zone. Adult specimens of mosquitoes, sand flies, biting midges and hard ticks were gathered from three forested areas that varied in their levels of anthropogenic disturbance and original habitat quality. These included a pristine site, Barro Colorado Island (BCI), which is comprised of old-growth forest with low levels of disturbance (e.g., >65% forest cover). In addition, two disturbed forest sites, Achiote (ACH) and Las Pavas (PVAS), encompass patches of secondary-growth forest subject to intermediate and high levels of disturbance (e.g., >35% and <65% forest cover) respectively [55,56]. Dipterans were collected using six Center for Disease Control (CDC) miniature light traps (John W. Hock Company, Gainesville, Florida), operating overnight in the understory (1.5 m height) and six in the canopy (> 25 m height), alternating each night. Each trap was situated along a transect and spaced at least 300 meters apart from each other to avoid pseudoreplication as in Loaiza *et al*. [55,57]. They were baited with 0.5 pounds of dry ice to attract blood-seeking dipterans. Adult specimens were retrieved from the traps at sunrise and taken to the laboratory in a portable freezer container holding dry ice. Individuals were sorted and identified using a chill table and taxonomic keys [58–62].

Ixodid ticks were collected with two methods at BCI and PVAS: the standard tick-dragging technique [63], and a pair of home-made cloth-pants, fabricated with white rustic fabric. Two human collectors traversed linear transects of up to 200 meters through the vegetation using either method. Adult specimens were removed from the cloth with entomological forceps, while immature stages (e.g., larvae and nymphs) were detached using transparent adhesive tape. Individuals were placed in separate cryo-vials, and subsequently transported to the laboratory. Taxonomic characters were used to identify ticks to the species level [64,65]. The samples were washed with 70% ethanol to remove surface contamination before storage in 95% ethanol. Details on the number of samples processed from each site and for each species are provided in S1 Table.

### DNA extraction, 16S rRNA gene library and sequencing

Each arthropod species was processed using the following laboratory procedures independently. Each sample was rinsed in 70% ethanol before they were pooled. DNA was isolated from pools of adult female dipterans and both adults and immature ticks using a BioSprint 96 robot and associated BioSprint® 96 DNA Blood kit (Qiagen, Gaithersburg, MD, USA). Each pool was crushed individually in tissue lysis buffer using a high-speed shaking TissueLyser II and ceramic beads; the supernatant was placed in a well of a 96-well plate and followed by DNA isolation protocol from the manufacturer. DNA pools were made by combining 2 μl of DNA extract from 20 to 35 individuals of sand flies and biting midges, plus up to 5 individuals per pool of mosquitoes and ticks. Pooled DNA was used as a template to amplify the V4 region of the 16S rRNA locus using a two-step PCR protocol. The first PCR was composed of 5 μl of 2X Maxima HotStart PCR Master Mix (Thermo), 0.2 μl of each primer (which included an Illumina sequencing primer on the 5’ end (10 mM)), and 1 μl of pooled DNA. Then 1 μl of the resulting PCR product was used to add on unique barcodes and Illumina sequencing adaptors in a second PCR of six cycles. The PCR cycling conditions had an initial denaturation step of 3 min at 94° C proceeding 25 cycles of 94°C for 45 sec, 50°C for 60 sec, and 72°C for 90 sec, followed by 10 min at 72°C extension. Resulting reactions were cleaned using PCR Normalization plates (Charm Biotech, San Diego, CA, USA) and samples pooled into a library which we concentrated using Kapa magnetic beads. The DNA concentration of each library was verified with the Qubit HS assay (Invitrogen, Waltham, MA, USA) and quality checked with a Bioanalyzer dsDNA High Sensitivity assay before sequencing on an Illumina MiSeq in a 2x250 paired end run. In the Culicidae family (mosquitoes), 40 pools of adult *Culex* including 20 pools of each *Culex coronator* and *Culex declarator* plus 20 pools of *Coquillettidia venezuelensis* were sequenced. Within the Ceratopogonidae (biting midges) and Psychodidae (sand flies), 94 pools of adult *Culicoides* including 34 pools of *Culicoides batesi*, 30 of *Culicoides foxi*, and 30 of *Culicoides heliconiae*, plus 75 pools of adult *Lutzomyia* including 30 pools of *Lutzomyia panamensis*, 23 of *Lutzomyia gomezi* and 22 of *Lutzomyia trapidoi* were sequenced and analyzed. Sequences within the hard tick family Ixodidae were obtained from 37 pools in total, including 6 pools of adults and 12 pools of nymphs of *Haemaphysalis juxtakochi*, 12 pools of adult *Amblyomma tapirellum* and 7 pools of adult of *Amblyomma oblongoguttatum* (S1 Table).

### Analysis of 16S metadata

Analysis of sequence reads was performed using the Quantitative Insights Into Microbial Ecology (QIIME) software package versions 1.9.1 and 2.0. The DADA2 data quality filtering pipeline implemented in QIIME 2.0 was used to trim sequences with base quality scores lower than 20. Operational taxonomic units (OTU’s) were assigned with a Naive Bayes classifier trained on the Greengenes 99% sequence similarity database v13.8 with sequences bound by the 515F and 806R primer pair [66]. Low abundance OTU’s (0.005%) were filtered from the resulting relative abundance table to reduce bias by sequencing error.

The feature table was rarefied to a sequencing depth of 7 000 reads before alpha and beta diversity values were calculated. The statistical test PERMANOVA was applied to the resulting UNIFRAC distance matrixes to test for significant differences between the beta diversity of metadata groups. Principle coordinates analysis (PCoA) plots were generated from unweighted UNIFRAC distance matrixes. In addition, taxonomic summary plots of the relative abundance of bacteria were generated to depict the bacterial orders with an overall proportion of > 0.1% in at least one species. Indicator species analysis was applied to identify the OTU’s unique to each species group.

## Results

In total, 11 435 639 sequence reads of the bacterial 16S gene were captured from 265 sample pools, encompassing 4 916 individuals from four different hematophagous arthropod families, six genera and 12 species. After quality filtering and rarefaction to a depth of 7 000 reads, 10 838 632 sequences remained from 229 sample pools with an average of 40 900 sequences per pool (SE ± 1,209) and a total of 1 404 OTU’s composed of 13 phyla, 30 classes, 55 orders, 106 families and 137 genera. Rarefaction curves revealed that the majority of bacterial diversity for all the species of arthropods was captured with subsampling of 7 000 sequences per sample pool (S1 Fig).

### Bacterial diversity and composition in mosquitoes, biting midges, sand flies and hard ticks

Among dipterans, members of the genera *Culex*, *Coquillettidia*, *Culicoides* and *Lutzomyia* had comparable proportions of bacterial OTU’s, bacterial diversity and community evenness index. In contrast, two tick species in the genus *Amblyomma* (i.e., *Amblyomma tapirellum* and *Amblyomma oblongoguttatum*) had higher number of OTU’s, and bacterial diversity, and the least even community composition. A third tick species, *Haemaphysalis juxtakochi*, had the highest overall bacterial phylogenetic diversity, although it had a lower number of OTU’s per tick pool and values of Shannon’s diversity compared to *Amblyomma* species (Table 1 and S1 Fig).

**Table 1:** Average measures of bacterial alpha diversity for 12 species of blood-feeding arthropods at a rarefaction depth of 7 000 16S sequences.

Taxonomy	Species		Observed OTU's	Shannon's diversity	Faith's phylogenetic diversity	Evenness
Acari:Ixodidae	*H*. *juxtakochi*		43.12	3.51	14.12	0.66
		**Adults**	55.71	3.39	11.65	0.69
		**Nymphs**	34.3	7.12	11.28	0.94
	*A*. *tapirellum*		194.82	7.15	11.43	0.95
	*A*. *oblongoguttatum*		144.75	5.64	11.5	0.81
Diptera:Culicidae	*Coq*. *venezuelensis*		53.35	3.67	5.83	0.65
	*Cux*. *coronator*		49.7	3.64	5.91	0.65
	*Cux*. *declarator*		49.2	3.29	6.39	0.59
Diptera:Ceratopogonidae	*C*. *batesi*		59.38	4.03	7.13	0.69
	*C*. *foxi*		61.73	3.93	7.18	0.67
	*C*. *heliconiae*		56.15	3.63	6.81	0.63
Diptera:Psychodidae	*Lu*. *gomezi*		55.04	2.99	7.1	0.52
	*Lu*. *panamensis*		62.97	3.59	6.85	0.61
	*Lu*. *trapidoi*		43.59	2.86	5.76	0.54

**Fig 1:**
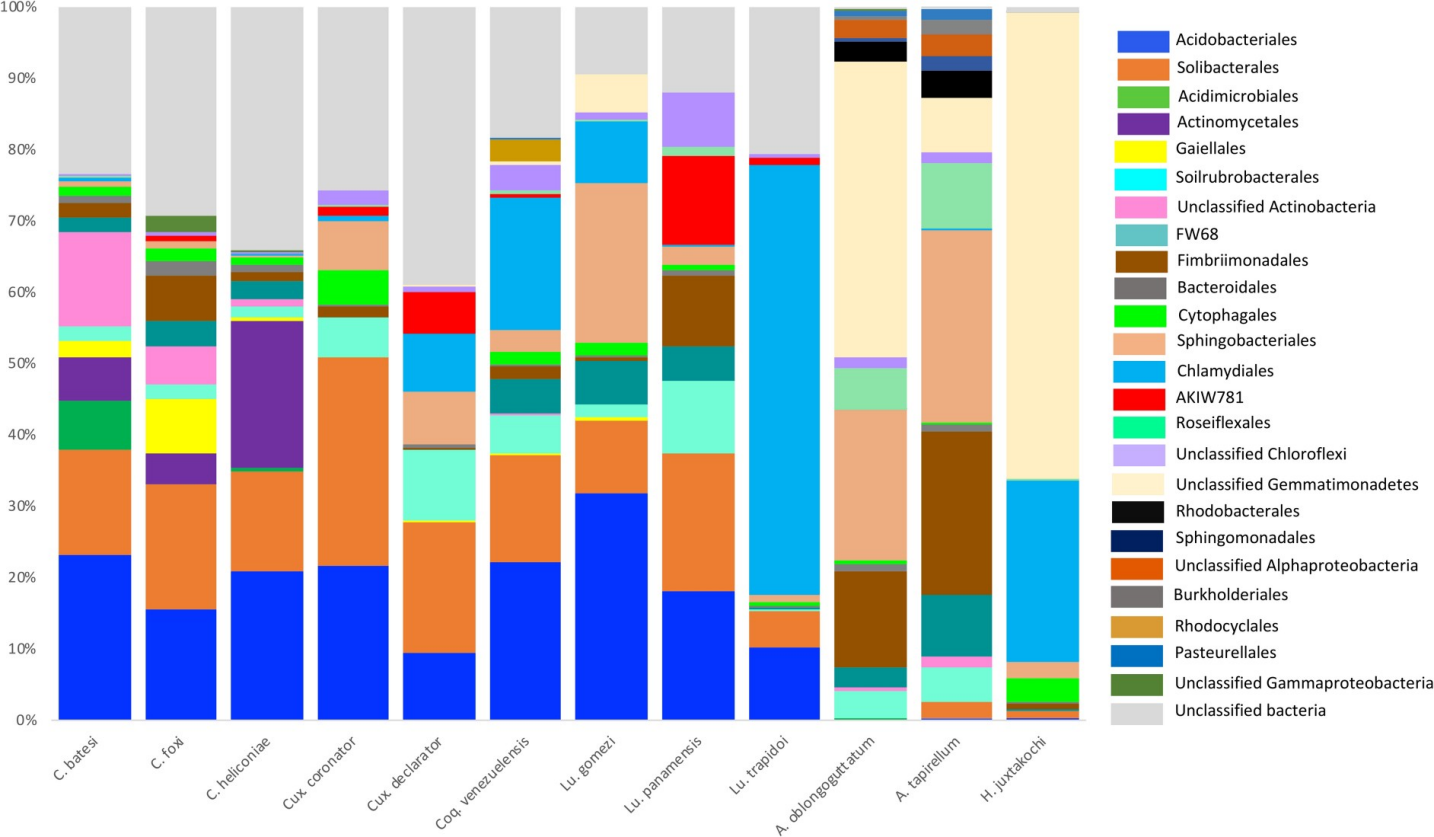
Relative abundances of bacterial orders above 0.1% summarized for each blood-feeding arthropod species.

All arthropod species were dominated by the phylum Proteobacteria with proportions ranging from 48 to 72%. Other major bacteria phyla that were shared among all arthropod species included Firmicutes, Bacteriodetes and Actinobacteria. Bacterial Orders and families were generally shared among arthropod genera in the Order Diptera, although they also exhibited notable differences in their relative proportions, which are visualized to the level of Order in Fig 1 and summarised to the genus level in S2 Table. Within the bacterial phyla shared between *Culex* and *Coquillettidia* mosquitoes, *Culicoides* biting midges, and *Lutzomyia* sand flies, the major classes consisted of Gammaproteobacteria, Betaproteobacteria, Alphaproteobacteria, Bacilli, and Actinobacteria.

*Culicoides* species share OTU’s with the other genera of dipterans, but PCA and taxonomic analysis revealed that they have a more distinct bacterial community than *Lutzomyia*, *Culex* and *Coquillettidia* together with unique bacterial types including a disease-causing agent in the genus Arcobacte*r* (proteobacterial class Epsilonproteobacteria, Order Campylobacterales) [67], and *Candidatus cardinium* (phylum of Bacteriodetes, class Cytophagia), which is known to alter arthropod reproduction [68]. Moreover, *Culicoides batesi*, *Culicoides foxi* and *Lutzomyia trapidoi* had unique OTU’s in the phyla Chlamydiae.

**Fig 2:**
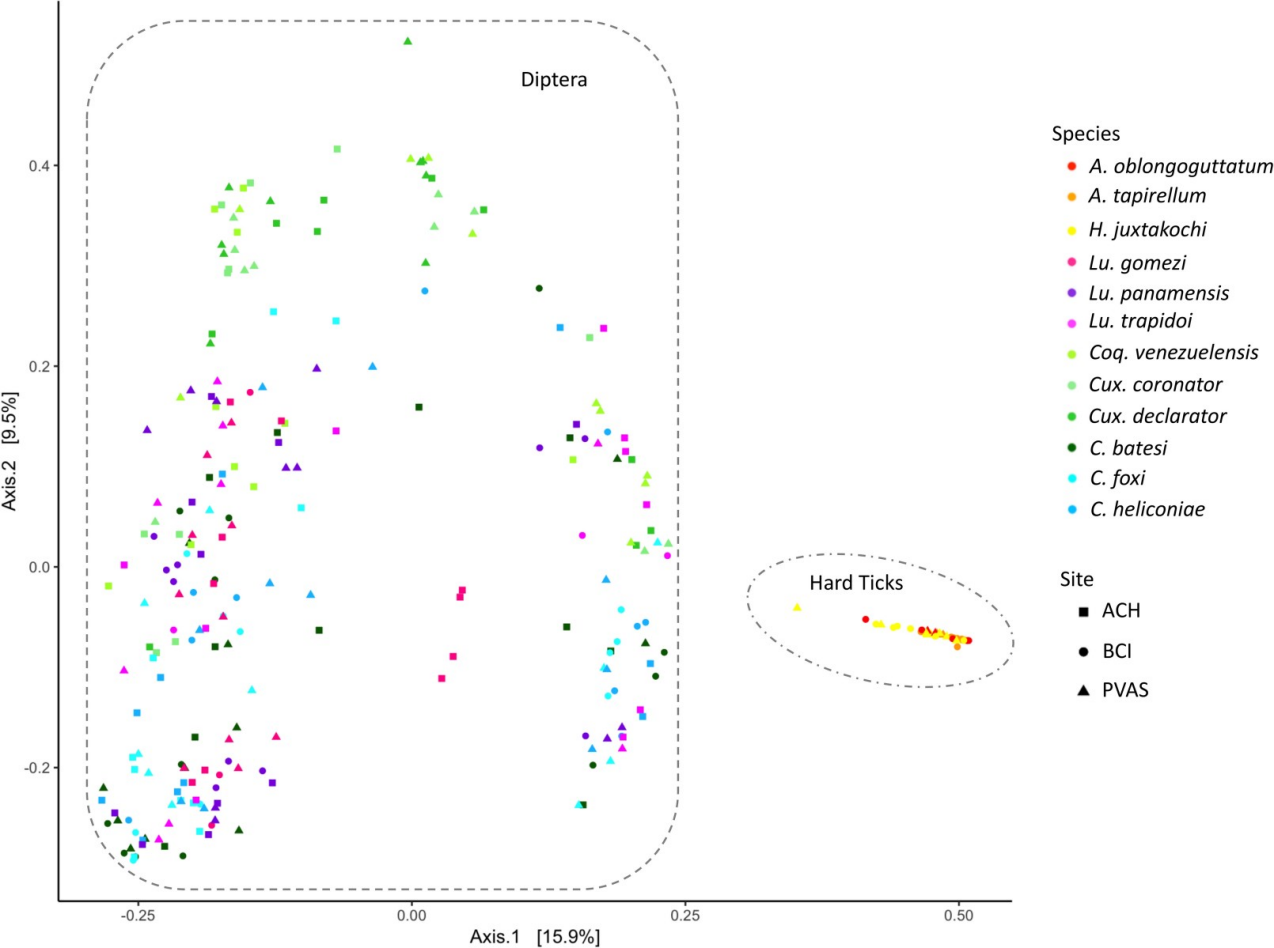
PCoA ordination analysis based on UNIFRAC distances with 16S gene sequence variation of the bacterial communities from six blood-feeding arthropod genera.

**Fig 3:**
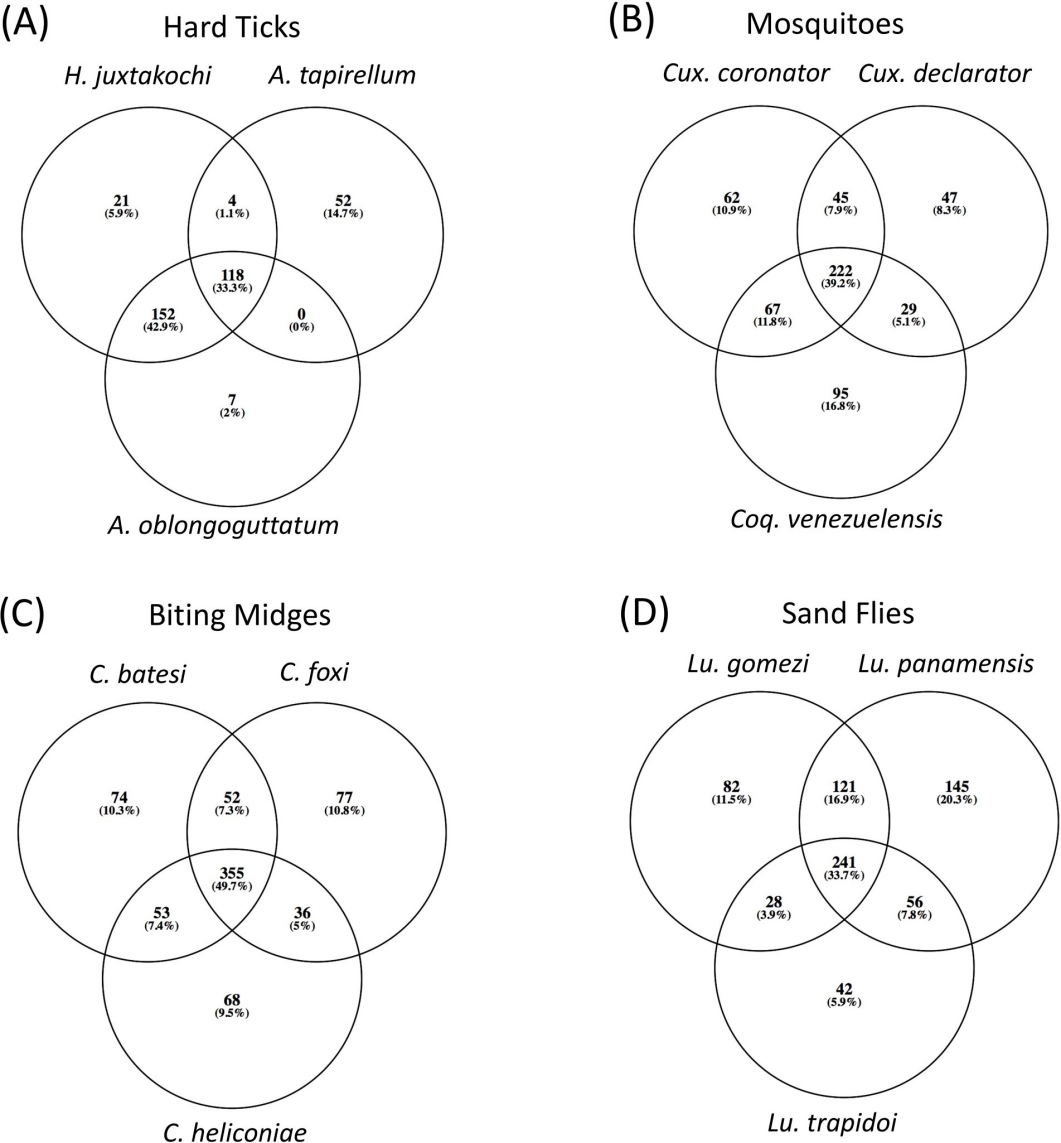
Venn diagram of shared and unique bacterial OTU’s among (a) three different species of Ixodidae; (b) two species of *Culex* (Culicidae) and one species of *Coquillettidia* (Culicidae); (c) three species of *Culicoides* (Ceratopogonidae); (d) three species of *Lutzmyia* (Psychodidae).

The bacterial phyla and classes of all tick species were composed of entirely different OTU’s than the other arthropod assemblages, hence they were the most distinct in terms of bacterial composition (Fig 2). Ticks in the genus *Amblyomma* had bacterial phyla that were not found in any other arthropod genus, including Chloroflexi, Acidobacteria, Gemmatimonadetes, Armatimonadetes and TM7. Likewise, *Amblyomma* ticks had a number of classes unique to this genus, including the Protobacterium Deltaproteobacteria, Saprospirae, Cytophagia within the phylum of Bacteriodetes, and the Actinobacteria Thermoleophilia and Acidimicrobiia. *A*. *tapirellum* had the largest proportion (14.7%) of OTU’s unique to its species (Fig 3).

All comparisons of the bacterial community among the different genera and species of adult hematophagous arthropods through PERMANOVA tests yielded statistically significant differences (Table 2). Additionally, there were no statistically significant differences between the adults and nymphs of *H*. *juxtakochi* based on UNIFRAC distances of bacterial OTU’s (PERMANOVA, pseudo-F = 1.38, P = 0.247), although they share only 80 OTU’s (25%). Variation in the number of OTU’s shared among the different arthropod species are visualized in Fig 3. Arthropods within the same genus shared between ~33 to 50% of OTU’s while a smaller proportion were unique to each species (between 2 to 20%). The taxonomy of indicator OTU’s for each arthropod species in this study identified as significant and with an indicator value over 0.25 are provided in S3 Table.

**Table 2:** Results of PERMANOVA test for the comparison of bacterial OTU’s among pools of six different genera and 10 different species (with within genera comparisons) of blood-feeding arthropods based on unweighted UNIFRAC distances.

Genera comparisons		No. of individuals within pools	No. of sample pools	pseudo-F	p-value	q-value
*Amblyomma*	*Coquilettidia*	195	39	77.076	0.001	0.001
*Amblyomma*	*Culex*	295	59	95.687	0.001	0.001
*Amblyomma*	*Culicoides*	2669	113	126.282	0.001	0.001
*Amblyomma*	*Haemaphysalis*	180	36	82.764	0.001	0.001
*Amblyomma*	*Lutzomyia*	2137	94	94.831	0.001	0.001
*Coquilettidia*	*Culex*	300	60	4.267	0.002	0.002
*Coquilettidia*	*Culicoides*	2674	114	17.305	0.001	0.001
*Coquilettidia*	*Haemaphysalis*	185	37	69.818	0.001	0.001
*Coquilettidia*	*Lutzomyia*	2142	95	10.184	0.001	0.001
*Culex*	*Culicoides*	2774	134	39.82	0.001	0.001
*Culex*	*Haemaphysalis*	285	57	86.494	0.001	0.001
*Culex*	*Lutzomyia*	2242	115	23.658	0.001	0.001
*Culicoides*	*Haemaphysalis*	2574	111	113.82	0.001	0.001
*Culicoides*	*Lutzomyia*	4616	169	25.43	0.001	0.001
*Haemaphysalis*	*Lutzomyia*	2127	92	85.509	0.001	0.001
**Species comparisons**						
*A*. *oblongoguttatum*	*A*. *tapirellum*	85	19	10.489	0.001	0.001
*Cux*. *coronator*	*Cux*. *declarator*	200	40	2.099	0.025	0.026
*C*. *batesi*	*C*. *foxi*	1825	64	1.499	0.102	0.102
*C*. *batesi*	*C*. *heliconiae*	1715	64	2.159	0.012	0.013
*C*. *foxi*	*C*. *heliconiae*	1608	60	1.611	0.064	0.065
*Lu*. *gomezi*	*Lu*. *panamensis*	1419	53	9.881	0.001	0.001
*Lu*. *gomezi*	*Lu*. *trapidoi*	1253	45	8.12	0.001	0.001
*Lu*. *panamensis*	*Lu*. *trapidoi*	1412	52	7.3	0.001	0.001
**Life Stage comparisons**						
*Haemaphysalis* adults	*Haemaphysalis* nymphs	85	17	1.38	0.247	0.247

### Effect of habitat disturbance on the organization of bacterial communities

**Fig 4:**
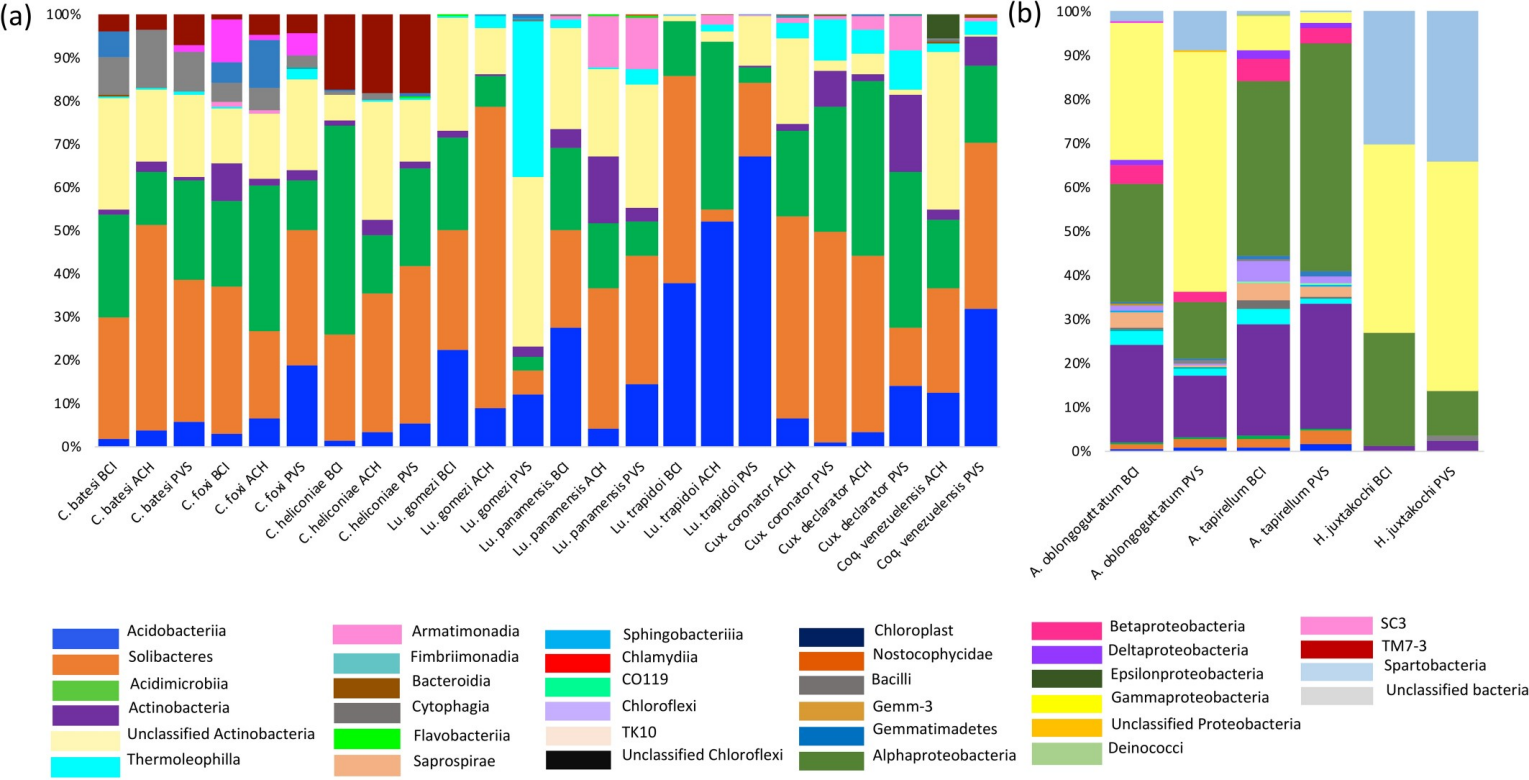
Relative abundances of bacterial classes summarized for (A) dipteran species and (B) hard ticks gathered from BCI (i.e., Pristine), ACH (i.e., intermediately disturbed) and PVAS (i.e., highly disturbed).

Intra-specific variation in the bacterial community was observed between sampling areas depicting different degrees of habitat disturbance for *Coquillettidia*, one *Culex* species, and all but one comparison of *Lutzomyia*, while another comparison between *Culex coronator* was close to significant (Table 3). Although bacterial diversity was comparable across pristine and disturbed habitats for most groups overall (S4 Table), we observed differences in the proportions of a number of bacterial types between pristine and disturbed habitats, although their associations differed within and among arthropod genera and species (Fig 4). For example, there was a high proportion of *Cyanobacteria* in both *Coquillettidia* and *Lutzomyia* from the disturbed sites at ACH and PVAS as well as an increased proportion of *Chlamydiae* for both *Culex* and *Lutzomyia* from the most disturbed site at PVAS. Similarly, there was an increased proportion of *Betaproteobacteria*, Order *Burkholderias* and the *Flavobacteriia*, family *Blattabacteriaceae* in pools of *Culex* from PVAS. Proportions of *Actinobacteria*, *Bacteriodetes*, *Flavobacteria* and *Bacteroidia* increased in *Luztomyia* from disturbed sites, whereas the proportion of *Deltaproteobacteria* increased from the pristine site BCI. Moreover, a number of bacterial classes including Nostococidae, Deltaproteobacteria, Deincoccci, Cytophagia and Chloroplast were found in *Coquillettidia* from the intermediately disturbed site at ACH, but not in the most disturbed site at PVAS. In contrast, within the three species of ticks, there was no difference in the proportion of bacterial classes between sampling areas or sampling method (Table 3, S4 Table and Fig 4). Similarly, no strong differences were detected in the bacterial classes of *Culicoides* among sampling areas or between vertical strata (i.e., forest understory or canopy) (Table 3 and S5 Table).

**Table 3:** Results of PERMANOVA test for the comparison of bacterial communities in pools of twelve different blood-feeding arthropod species among sampling areas based on unweighted UNIFRAC distances and 999 permutations. Significant results are highlighted in bold.

Taxonomy	Species	Site comparison		No. of sample pools	pseudo-F	p-value	q-value
Diptera:Culicidae	*Coq*. *venezuelensis*	ACH	PVS	20	6.920	**0.001**	**0.001**
	*Cux*. *coronator*	ACH	PVS	20	2.042	0.055	0.055
	*Cux*. *declarator*	ACH	PVS	20	3.061	**0.001**	**0.001**
Diptera:Ceratopogonidae	*C*. *batesi*	ACH	BCI	23	1.797	0.092	0.100
		ACH	PVS	22	2.229	0.027	0.081
		BCI	PVS	23	1.529	0.100	0.100
	*C*. *foxi*	ACH	BCI	20	1.627	0.087	0.131
		ACH	PVS	20	1.363	0.196	0.196
		BCI	PVS	20	1.882	0.041	0.123
	*C*. *heliconiae*	ACH	BCI	15	1.120	0.356	0.534
		ACH	PVS	15	2.104	0.041	0.123
		BCI	PVS	10	0.868	0.630	0.630
Diptera:Psychodidae	*Lu*. *gomezi*	ACH	BCI	13	5.341	**0.005**	**0.006**
		ACH	PVS	20	2.709	**0.002**	**0.006**
		BCI	PVS	13	3.311	**0.006**	**0.006**
	*Lu*. *panamensis*	ACH	BCI	20	3.033	**0.002**	**0.005**
		ACH	PVS	20	1.581	0.070	0.070
		BCI	PVS	20	2.645	**0.003**	**0.005**
	*Lu*. *trapidoi*	ACH	BCI	13	2.936	**0.004**	**0.005**
		ACH	PVS	19	3.107	**0.002**	**0.005**
		BCI	PVS	12	3.458	**0.005**	**0.005**
Acari:Ixodidae	*H*. *juxtakochi*	BCI	PVAS	17	0.254	0.918	0.918
	*A*. *tapirellum*	BCI	PVAS	12	1.584	0.157	0.157
	*A*. *oblongoguttatum*	BCI	PVAS	7	2.006	0.133	0.133

## Discussion

Habitat disturbance resulting from land use change can alter arthropod-borne disease transmission dynamics by modifying the habitat characteristics, community composition, behaviour, and patterns of dispersal and distribution of vectors or hosts [55,69,70]. Furthermore, habitat disruption can also modify the bacterial composition of natural environments, such as in the case of soil microbiota [71]. Yet, to our knowledge, no study has looked at the influence of habitat disturbance on the microbiome of human disease vectors, especially those that develop and interact with bacteria in the water, leaf litter, and soil or are acquired through animal host feeding in ecologically altered areas.

We tackled this issue by assessing bacterial communities associated with blood-feeding arthropods across sites with different degrees of habitat disturbance in the lowland tropical rainforest of central Panama. Specifically, we applied a 16S gene bacterial metagenomic approach to evaluate whether variation in the microbiome is associated with taxonomic relatedness, habitat disturbance or a combination of both. We focused on adults of Culicidae mosquitoes (i.e., *Culex* and *Coquillettidia*), Psychodidae sand flies (i.e., *Lutzomyia*) and Ceratopogonidae biting midges (i.e, *Culicoides*), which share ecological similarities in their development and adult life stages. Both *Culex* and *Coquillettidia* mosquitoes develop in aquatic sites associated with the roots of floating plants, while members of *Culicoides* develop in damp soil, water and organic matter [13,72–74]. All species of *Lutzomyia* develop in the soil within dark and humid places such as burrows and crevices associated with abundant leaf-litter or decomposing organic matter [75]. The males of *Culex*, *Coquillettidia*, *Culicoides* and *Lutzomyia* feed on nectar while the females take blood from a wide range of bird and mammal hosts. In addition, we sampled both nymphs and adult Ixodidae (i.e., *Amblyomma*, *Ixodes*, *Haemaphysalis*), which are distinct in their ecology compared to dipterans. Both the nymphs and adults of hard ticks adhere to and feed on vertebrate hosts throughout their lifetime [76]. Although they spend time off their host to molt through the different life stages and “quest” for a new host, they do not depend on these environments for feeding.

Our results are generally similar to those obtained in previous studies, where arthropod vectors species were dominated by Proteobacteria, including *Gammaproteobacteria*, *Betaproteobacteria*, *Alphaproteobacteria*, and to a lesser extent by Firmicutes, commonly *Bacilli* and *Actinobacteria* [13,18,44–46,77,78], These groups included bacterial genera previously described for *Culex* [13,44,46,77], *Culicoides* [21,38], *Lutzomyia* [37,79], *Haemaphysalis* [78,80] and *Amblyomma* [42,81].

We found that mosquitoes, biting midges and sand flies share a large proportion of their bacteria but statistical analysis also revealed significant differences in the OTU composition of each genera and species. It should be noted that variability at the 16S rRNA region, primer affinity and composition of the bacterial database will influence the resolution of the between-species comparisons based on OTU’s [82]. However, this finding suggests that these arthropods might encounter distinct bacterial types associated with differences in their habitat use or diet. Also, the colonization success of these bacterial types could differ among the arthropod hosts. We found that all tick species shared some bacterial OTU’s, but that this association did not extend to the dipteran assemblages. This is likely to reflect both their degree of taxonomic relatedness, since phylogenetically related species tend to share similar functional microbiomes [83], but also their distinct ecology. For instance, while all dipteran genera undergo larval development in either aquatic sites or organic soil before blood feeding as adults, hard ticks are largely associated with their host throughout their lifetime. Ticks undergo a series of molting events after each blood meal, which could be obtained from a series of animal hosts, from which they are expected to acquire much of their microbiome [80], while some symbiotic bacteria are also maternally inherited [2]. In contrast, dipteran genera also acquire bacteria through blood feeding, but their microbial community maintained through to adulthood is largely acquired during larval feeding and contact with the physical environment [13,48,49].

We observed significant differences in the bacterial community among areas with different degrees of habitat disturbance for two ecologically similar mosquito species within *Culex* and *Coquillettidia*, and three *Lutzomyia* sand fly species. These differences could be related to changes in the mammal or bird communities that served as feeding choices for adult arthropods as a result of habitat disruption. Alternatively, intra-specific differences could also result from changes to the pool of environmental bacteria, which might be associated with habitat disturbance. In support of these assumptions, we observed differences in a number of environmentally associated bacteria between primary forest, secondary forest and agricultural land, although changes in specific bacterial types generally vary among the different arthropod assemblages. For instance, the *Cyanobacteria nostococidae*, which has previously been associated with aquatic environments inhabited by mosquito larvae [13], was present in both *Culex* and *Lutzomyia* collected from secondary forest and disturbed habitats, but not from pristine forest sites. In addition, it was more common for *Culex* and *Lutzomyia* to be associated with *Chlamydia* in secondary forest and disturbed pastureland than in pristine forest, suggesting either differences in the mammal host reservoir or increased infection of mammals associated with changes in habitat quality.

We did not observe a significant difference in the bacterial community for any *Culicoides* species as a function of habitat disturbance. A potential explanation for this outcome is that *Culicoides* species either share a narrow ecological niche or because their optimal breeding habitats are not impacted by habitat disturbance. *Cuilicoides* regularly develop in areas with a high degree of organic matter known to modulate bacterial diversity [84], and are sensitive to temperature and humidity [85]. Nonetheless, the bacterial community of *Culicoides* in their preferred breeding sites has thus far been poorly characterized. Characterization of the differences in microhabitat features in *Culicoides* between land use types is required to confirm whether their breeding habitats and associated microbiota remain stable despite habitat disturbance. Furthermore, the host preferences of *Culicoides*, including the species in the current study are poorly classified and generally unknown within natural habitats, but some studies showed that most *Culicoides* species are opportunistic feeders, while others specialize on birds or mammals [86,87]. Another explanation for the lack of differences in the bacterial community of *Culicoides* between sites could be a stricter association of bacteria with the insect host than for other dipterans. That we did not see significant intra-specific differences in the bacterial community among tick species across areas with different habitat quality is not surprising given their specialized ecology [88].

We identified OTUs of several disease-causing bacteria as well as bacteria thought to alter life history characteristics and/or viral replication in all the arthropod genera, although these could not be identified to species. For example, we amplified *Coxiella*, whose members cause Q fever from all three tick species, *Ehrlichia* which causes ehrlichiosis infection from *A*. *tapirellum* and *Rickettsia* from *A*. *oblongoguttatum* and *H*. *juxtakochi*, which causes a variety of bacterial infections in humans and animals [89]. In addition, *Rickettsia* was also identified from *Lu*. *trapidoi* while *Bartonella* was detected from *Lu*. *panamensis* and *Lu*. *gomezi* plus all three species of *Culicoides*.

*Rickettsia rickettsii*, known to cause Rocky Mountain spotted fever in Panama has been previously isolated from *Amblyomma mixtum*, *Dermacentor nitens* and *Haemaphysalis leporispalustris*. In addition, two other *Rickettsia* species have been isolated from ticks in Panama including *Rickettsia bellii* from *Amblyomma rotundatum* and *Rickettsia amblyommii* from *A*. *mixtum* [90]. Although identification of the *Rickettsia* OTU’s were not to species level in this study, to our knowledge, this is first record of *Rickettsia* isolated from *A*. *oblongoguttatum* and *H*. *juxtakochi* in Central America as well as from *Lutzomyia spp*. However, agents causing bartonellosis have not yet been described from *Culicoides* biting midges. The ability of *Culicoides* to vector *Bartonella* requires further confirmation, but its presence in all three species is suggestive of a likely transmission role in Panama.

Congruently, we found several genera of bacteria with the potential to impact vector pathogen transmission. For instance, the genus *Paenibacillus*, which can inhibit DENV replication in *Aedes* mosquitoes was present in all *Culicoides* species as well as in *Lu*. *panamensis* [28]. Similarly, *Serratia* which can increase DENV and CHIKV in *Ae*. *aegypti* mosquitoes was present in all species of biting midges, mosquitoes and sand flies [28]. The family *Enterobacteriae*, which has been known to increase *Plasmodium* parasite infection in *Anopheles* mosquitoes was present in all, but *A*. *oblongoguttatum* [36]. Moreover, the bacteria *Wolbachia*, which impacts on vectors of arboviruses, *Plasmodium* infection and life history traits such as reproductive fitness and adult lifespan [91–94] was found from all Diptera.

## Conclusion

Habitat disturbance has been shown to increase the likelihood of disease outbreaks of zoonotic (e.g., animal origin) infections through modifying the vector or host communities, or impacting their life history characteristics. However, the epidemiological role of bacteria associated with blood-feeding arthropods in relation to habitat disturbance is still poorly understood. Here, we observed that variation in the bacterial communities across a diverse array of hematophagous arthropods is likely to be explained by host phylogenetic relatedness, while intraspecific changes in community composition and prevalence are influenced by habitat quality. We found that the proportions of known disease-causing agents in infected arthropod species were comparable across sampling areas with different levels of habitat disturbance. However, further work is needed to determine whether the changes to the bacterial community with habitat disruption could influence disease transmission to humans. We argue further that changes in the microbiome of disease vectors should be considered when assessing the impact of habitat disturbance on disease transmission risk and emergence.

## Supporting information

S1 Fig Rarefaction results based on Faith’s Phylogenetic Diversity of 12 species of blood-feeding arthropods at a rarefaction depth of 7 000 16S rRNA sequences per species. (TIF)

S1 Table Collection and processing information for each pool of arthropod samples. (XLSX)

S2 Table Relative proportions of bacteria for each arthropod species and composite individuals. (XLSX)

S3 Table The bacteria identified by indicator species analysis as significant for each arthropod species. (XLSX)

S4 Table Results of PERMANOVA test for the comparison of bacterial OTU’s in pools of three Ixodid species between sampling methods based on unweighted UNIFRAC distances and 999 permutations. (XLSX)

S5 Table Results of PERMANOVA test for the comparison of bacterial communities in pools of three *Culicoides* species originating from the ground or canopy level collections based on UNIFRAC distances. (XLSX)
