# Correction: Habitat disturbance and the organization of bacterial communities in Neotropical hematophagous arthropods

**DOI:** 10.1371/journal.pone.0225226

**Published:** 2019-11-07

**Authors:** Kelly L. Bennett, Alejandro Almanza, W. Owen McMillan, Kristin Saltonstall, Evangelina López Vdovenko, Jorge S. Vinda, Luis Mejia, Kaitlin Driesse, Luis F. De León, Jose R. Loaiza

Figs 1 and 4A are incorrect. The authors have provided corrected versions here.

**Fig 1:**
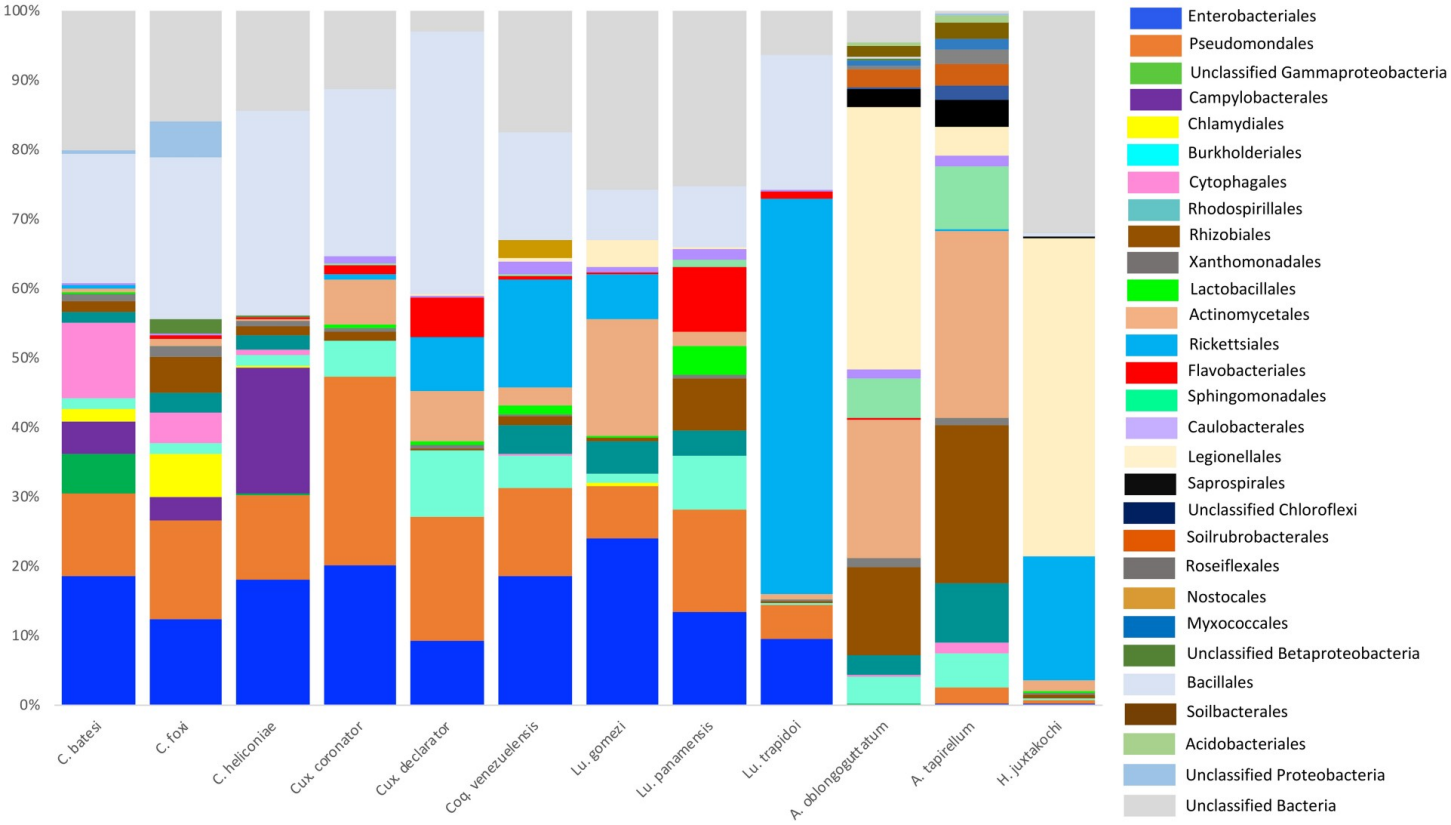
Relative abundances of bacterial orders above 0.1% summarized for each blood-feeding arthropod species.

**Fig 4:**
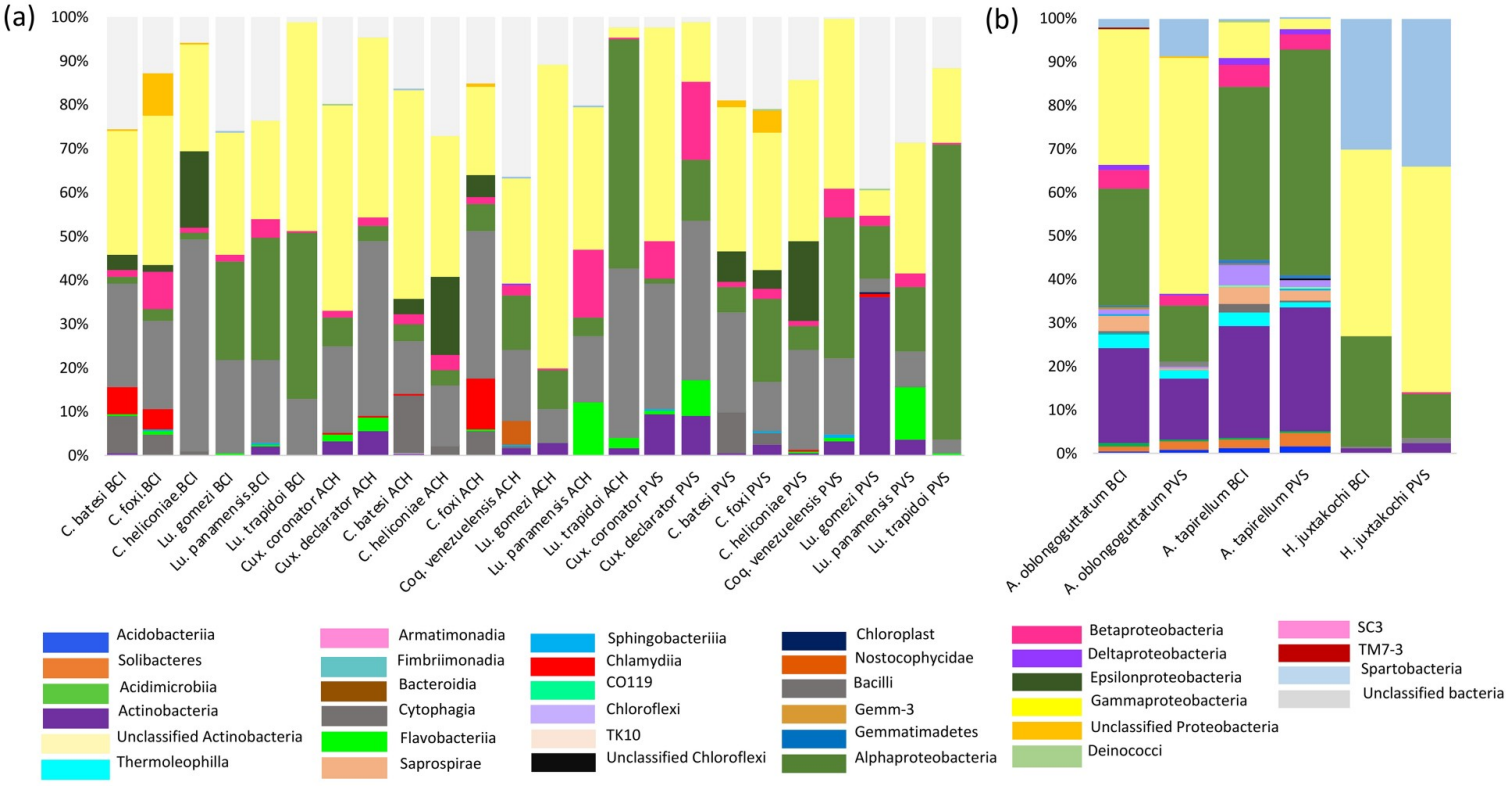
Relative abundances of bacterial classes summarized for (A) dipteran species and (B) hard ticks gathered from BCI (i.e., Pristine), ACH (i.e., intermediately disturbed) and PVAS (i.e., highly disturbed).
