# Supplementary figures and images for: Habitat disturbance and the organization of bacterial communities in Neotropical hematophagous arthropods
Source: PLoS One. 2019 Sep 6;14(9):e0222145. doi: 10.1371/journal.pone.0222145 (PMC6730880; doi:10.1371/journal.pone.0222145)

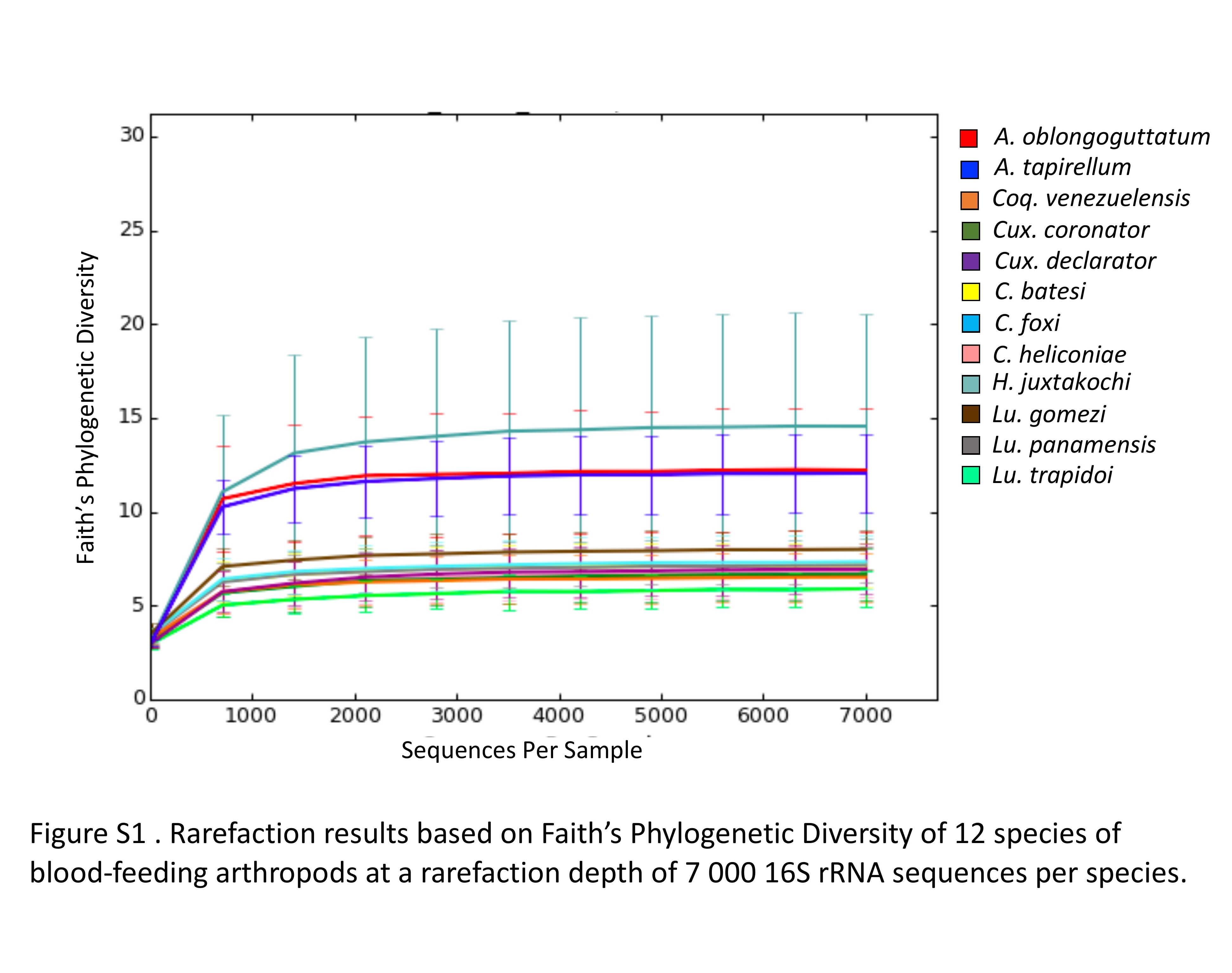

Supplement: S1 Fig — (TIF) [file pone.0222145.s001.tif]
